# Information Theory Based Evaluation of the RC4 Stream Cipher Outputs

**DOI:** 10.3390/e23070896

**Published:** 2021-07-14

**Authors:** Evaristo José Madarro-Capó, Carlos Miguel Legón-Pérez, Omar Rojas, Guillermo Sosa-Gómez

**Affiliations:** 1 Facultad de Matemática y Computación, Instituto de Criptografía, Universidad de la Habana, Habana 10400, Cuba; ejmcapo@gmail.com (E.J.M.-C.); clegon58@gmail.com (C.M.L.-P.); 2 Facultad de Ciencias Económicas y Empresariales, Universidad Panamericana, Álvaro del Portillo 49, Zapopan 45010, Jalisco, Mexico; gsosag@up.edu.mx

**Keywords:** RC4, iterative probabilistic attacks, entropy, randomness

## Abstract

This paper presents a criterion, based on information theory, to measure the amount of average information provided by the sequences of outputs of the RC4 on the internal state. The test statistic used is the sum of the maximum plausible estimates of the entropies $H(j_{t}|z_{t})$, corresponding to the probability distributions $P(j_{t}|z_{t})$ of the sequences of random variables ${\left(j_{t}\right)}_{t\in T}$ and ${\left(z_{t}\right)}_{t\in T}$, independent, but not identically distributed, where $z_{t}$ are the known values of the outputs, while $j_{t}$ is one of the unknown elements of the internal state of the RC4. It is experimentally demonstrated that the test statistic allows for determining the most vulnerable RC4 outputs, and it is proposed to be used as a vulnerability metric for each RC4 output sequence concerning the iterative probabilistic attack.

## 1. Introduction

In [1], the iterative probabilistic attack was proposed to reconstruct the internal state of the RC4 algorithm, starting from knowing an output sequence, which was successively improved in [2,3]. In essence, these attacks attempt to extract information about the content of the internal state $\left\{\left(j_{t},\;S_{t}\right):t=1,\ldots ,T\right\}$ of the algorithm RC4 stream cipher from a known output sequence $\left\{\left(z_{t}\right):t=1,\ldots ,T\right\}$. For this, the conditional probabilities $P(j_{t}|z_{t})$ and $P(S_{t}|z_{t})$ are iteratively recalculated. This type of attack does not yet violate RC4, but it constitutes a serious potential threat to its security, which should not be ignored. Concerning this threat, a criterion has been developed to assess the vulnerability of an RC4 output to this type of attack. The test statistic used is based on the entropy of the conditional probability distributions $P(j_{t}|z_{t})$ for the $z_{t}$ that appear in the evaluated sample. The test statistic was proposed considering that the values and position of these $z_{t}$ determine their probability distribution and associated entropy. The lower the value of the statistic, the more vulnerable the evaluated sample is the lower the attacker’s uncertainty will be about the value of the variable $j_{t}$.

This result can have various applications, since it allows for an evaluation of a set of RC4 output sequences according to their vulnerability or theoretical strength in face of iterative probabilistic attacks. This criterion can characterize the keys that cause the greatest vulnerability, which can lead to the identification of a new class of weak keys. In this work, experimental results evaluating the RC4 output sequences, according to their vulnerability to probabilistic attacks, are presented.

The structure of this work is as follows: in Section 2 the basic concepts of the research topic are described, which includes the description of the RC4 algorithm and the reports associated with the iterative probabilistic attack; Section 3 introduces the statistic used to evaluate the vulnerability of the RC4 outputs concerning the iterative probabilistic attack; Section 4 details the pre-calculation of frequencies that allow the estimation of the joint, marginal, and conditional probabilities and, in turn, the estimation of the entropies that will be used for the statistical calculation on the output sequences of RC4; in Section 5 experiments are performed to validate the proposed statistician. The results of applying the statistic on RC4 output sequences are illustrated; finally, Section 6 presents some conclusions.

## 2. Preliminaries

### 2.1. Description of the RC4 Stream Encryption Algorithm

The RC4 algorithm [4] stands out from other stream ciphers for its wide use in different applications and protocols. The RC4 stream cipher [4] is optimized to be used in 8-bit processors, being extremely fast and exceptionally simple. It was included in network protocols, such as secure sockets layer (SSL), transport layer security (TLS), wired equivalent privacy (WEP), Wi-Fi protected access (WPA), and in various applications used in Microsoft Windows, Lotus Notes, Apple Open Collaboration Environment (AOCE), and Oracle Secure SQL [4]. In the last decade, some applications [5,6] have avoided RC4 encryption, given some found weaknesses [7]. However, although it is not considered very secure [8], RC4 continues to motivate research nowadays [8,9,10]. Furthermore, this cipher is a good option to measure the effectiveness of methods that analyze weaknesses in stream ciphers related to those already known in RC4 [11,12,13,14], or to check the performance of hardware or software schemes that make use of cryptography [15,16,17].

The RC4 has two main components, the key scheduling, and the pseudorandom number generator. The key scheduling generates an internal random permutation *S* of values from 0 to 255, from an initial permutation, a (random) key *K* of *l*-byte length, and two pointers *i* and *j*. The maximal key length is of l=256 bytes, see Algorithm 1.
**Algorithm 1** RC4 key-scheduling.1:**for**i=0→255**do**2:    S[i]←i3:**end for**4:j←05:**for**i=0→255**do**6:    j←(j+S[i]+K[imodl])modn7:    Swap S[i] and S[j]8:**end for**

The main part of the algorithm is the pseudo-random number generator that produces one-byte output at each step. As usual, for stream ciphers, the encryption will be a XOR of the pseudo-random sequence with the message, see Algorithm 2.
**Algorithm 2** RC4 pseudo-random generator.1:i←02:j←03:**while** Generating Output **do**4:    i←(i+1)mod2565:    j←(j+S[i])mod2566:    Swap S[i] and S[j]7:    Output S[(S[i]+S[j])mod256]8:**end while**

For the RC4 stream cipher, several modifications have been proposed, while some modified only certain components or some operations, others completely changed the algorithm, see [18]. It is important to note that even RC4 variants have had a lot of attention in the scientific community, see [19].

The RC4 stream cipher, in its definition, does not distinguish the use of IV initialization vectors [4]. However, it is well known that in practical applications of RC4, as in many other stream ciphers, an IV initialization vector with a secret key is used to form a session key. The proposed method is independent of the approach used; it simply works on the final input used as input to the cipher.

### 2.2. Iterative Probabilistic Attacks

Here we discuss three important results on probabilistic attacks that try to reconstruct the internal state of RC4 from knowing an output sequence $\left\{\left(z_{t}\right):t=1,\ldots ,T\right\}$. In [1], the central idea, proposed by Knudsen et al., was to conveniently use Bayes’s Theorem to recalculate the probabilities $P(j_{t}|z_{t})$ and $P(S_{t}|z_{t})$, for each t∈T. In essence, they worked on obtaining probabilistic information about the two variables $j_{t},S_{t}$ from $z_{t}$. It was reported that a low probability of success and a high volume of work were achieved. To be successful, it was required to know the values of at least *d* elements of $S_{0}$, with d∈{150,…,160}. The results presented in [1] are independent of the key scheduling and the key size. For sequences of length $T=256=2^{8}$, the volume of work was $2^{48}$ in each iteration. In [2], Knudsen’s method was improved by reducing the number of elements of the permutation that must be known and maintains the same workload of $2^{48}$ in each iteration. The essential difference is that a more exact way of recalculating the probabilities was proposed using the entire *Z* output sequence instead of just the $z_{t}$ value to increase the probability of success. Experiments were reported for RC4 with n=3 and n=4. Finally, in [3], Golic and Morgari used the same probabilities of the previous article; the novelty in that work was that it proposed a set of 7 improvements to the probabilistic algorithm itself and estimated the minimum number *d* of elements in $S_{0}$ that must be known a priori so that the attack recovers the correct $S_{0}$ permutation, concluding that d∈{26,…,85}, which is a substantial improvement compared to d∈{150,…,160}. The workload remained at $2^{48}$ probabilities that must be calculated at each iteration.

In summary, in the three aforementioned articles, it was reported that these attacks have a low probability of success when no element of the permutation is known a priori, which is why it is concluded that they are not currently applicable to real RC4. In such articles, the authors model the ignorance over the internal state assuming the initial uniform probability distribution for *S* and *j*. It is essential to note that increasing the precision of the recalculated probabilities reduced the number *d* of elements of the permutation that must be known a priori. Knudsen et al. got d∈{150,…,160}, while Golic and Morgari reduced it to d∈{26,…,85}. The previous result suggests that by increasing the precision of the calculated probabilities in different ways or by improving the iterative algorithm, it could be possible to achieve d≈0, i.e., to recover the complete permutation without knowing any of its elements a priori, which constitutes a serious threat to the safety of the RC4.

### 2.3. Entropy As a Measure of Uncertainty

Let *X* be a discrete random variable with possible values $x_{i}$ and respective probabilities $p_{i}=P\left(x_{i}\right)$, with i=1,…,k. Then, Shannon’s discrete entropy function $H(p_{1},\ldots ,p_{k})$ [20] is defined as

(1) $$H\left(X\right)=-\sum_{i=1}^{k}p_{i}\log p_{i}.$$

When $p_{i}=1/k$ for all i=1,…,k, the maximum uncertainty about the value of *X* is obtained, so the entropy reaches its maximum value, equal to

(2) $$H_{max}=H\left(\frac{1}{k},\ldots ,\frac{1}{k}\right)=log\left(k\right).$$

When there is an $i^{\prime }$ such that $p_{i^{\prime }}=1$ and $p_{i}=0$ for all $i\neq i^{\prime }$, there is no uncertainty about the value of *X*, so the entropy reaches its minimum value, equal to $H_{min}=0$.

## 3. Definition of the Proposed Test Statistic

In this work, the information that $z_{t}$ contributes on $j_{t}$ probabilistically, by means of a non-uniform probability distribution, will be modeled from the knowledge of $z_{t}$. To support this proposal, we start from the relationship between $z_{t}$ and $j_{t}$, and the result of [4] on the non-equi-probability of the permutation *S* at the beginning of the pseudo random generation algorithm (PRGA) stage.

Solving for $j_{t}$ in the equation that defines $z_{t}$ in the RC4 algorithm, we obtain:

(3) $$j_{t}=S_{t}^{-1}\left[S_{t}^{-1}\left[z_{t}\right]-S_{t}^{-1}\left[i_{t}\right]\right].$$

For t=0, we have $j_{0}=S_{0}^{-1}\left[S_{t}^{-1}\left[z_{0}\right]-S_{0}^{-1}\left[i_{0}\right]\right]$. Note that the values of $i_{0}$ and $z_{0}$ are known, while $S_{0}$ is unknown, therefore, the distribution of $j_{0}$ is determined by $S_{0}$. Taking into account that in [4] it is shown that $S_{0}$ does not follow a uniform distribution, it is considered that for t=0, when $z_{0}$ is known, this property of non-uniformity is translated to $j_{0}$.

Expression (3) does not allow the calculation of $j_{t}$ since $S_{t}$ is unknown. However, it allows theoretically arguing the non-equi-probability of $j_{t},$ conditional on knowing the value of $z_{t}$ (due to the non-equi-probability of *S*).

Denoting by $t^{*}$ the smallest value of *t*, such that for $t>t^{*}$ it is true that $S_{t}$ follows a uniform distribution. In [21,22], the authors tried to estimate the value of $t^{*}$. From this definition, and following the same reasoning as for $S_{t}$, at t=0, the beginning of the first iteration; it can be assumed that for $t\in \{0,\ldots ,t^{*}-1\}$ the conditional probability distribution $P(j_{t}|z_{t})$, is non-uniform. In [22], it is described that it is possible to find biases in the output bytes of RC4 up to $t^{*}=512$. Thus, for t∈{0,…,511} the conditional probabilities $P(j_{t}|z_{t})$ do not fit a uniform distribution.

### 3.1. Basis of the Evaluation Criterion

The criterion will be limited to considering only the variable $j_{t}$ and its conditional probabilities $P(j_{t}|z_{t})$. This was taken into account because the knowledge without errors of the sequence $\left\{\left(j_{t}\right):t=1,\ldots ,T\right\}$ allows to reconstruct $S_{0}$ [5]. The central idea of the criterion is based on the different values $z_{t}$ that appear at each time step t∈T. This can cause different initial conditional probability distributions $P(j_{t}|z_{t})$ to appear at each t∈T. This is the essence of the proposed test statistic; i.e., it will take into account which values $z_{t}$ appear in the sample and in which places (times *t*) each one of those values $z_{t}$ appears. Under this condition, two samples with different frequency distributions $z_{t}$ will have different vulnerabilities to attack. Even between two samples with the same frequency distribution of the values $z_{t}$, the effectiveness would vary depending on their places of appearance.

### 3.2. Definition of the Test Statistic

To measure these differences, a 256×256 matrix was pre-calculated at each time t={1,…,T}, in which the columns represent all the possible values of $z_{t}=0,\ldots ,255$ and the rows each value of $j_{t}=0,\ldots ,255$. The element (j,z) of the matrix, corresponding to row *j* and column *z*, constitutes the conditional probability $P(j_{t}|z_{t})$, at time t∈T. In this way, each column will be the distribution of conditional probabilities $P(j_{t}|z_{t})$, which probabilistically represents the information about *j* that causes the appearance of *z* at this time. The most interesting question one might ask is: how can one compare two different columns? For example, how can one compare two probability distributions? More exactly, which distribution is associated with the greatest uncertainty about *j*? To solve this problem, the concept of Shannon’s discrete entropy will be used.

For each column, the entropy will be calculated, denoted by $H_{z}^{t}$, which is a direct measure of the uncertainty about $j_{t}$, when in the place *t* of the sample appears the $z_{t}$ value associated with that column. It is important to mention that the entropy value characterizes the distribution of the 256 possible values of $j_{t}$ in a single value, facilitating the comparison between probability distributions. By entropy’s properties, it is satisfied that if $H_{z}^{t}=0$, then the value of the $j_{t}$ variable is determined by $z_{t}$, while if $H_{z}^{t}=8$, knowledge of $z_{t}$ does not provide any information about the value of $j_{t}$.

To evaluate in a sample of length *T*, the total uncertainty over *j*, the entropy associated with the value $z_{t}$ that appeared at each time t=1,…,T will be added over all times. Then, the expression of the test statistic will be:

(4) $$Q=\sum_{t=0}^{T}H_{z}^{t},$$

where

(5) $$H_{z}^{t}=-\sum_{j_{t}=0}^{255}P\left(j_{t}|z_{t}\right)logP\left(j_{t}|z_{t}\right).$$

The expected value μ=E(Q) and the variance $\sigma^{2}=V\left(Q\right)$ of the statistic *Q*, are expressed from the expected value and the variance of the conditional entropies $H_{z}^{t}$ in the *T* times and are given by

(6) $$\mu =E\left(Q\right)=\sum_{t=0}^{T}E\left(H_{z}^{t}\right),$$

and assuming that the $H_{z}^{t}$, with t=1,…,T, are independent of each other,

(7) $$\sigma^{2}=V\left(Q\right)=\sum_{t=0}^{T}V\left(H_{z}^{t}\right).$$

For each entropy $H_{z}^{t}$ that appears as an addition in the expression of *Q*, its distribution can be approximated by a normal distribution according to the result of [23]. However, this plug-in estimator is known to be biased. Its bias and variance [24,25], are given by

(8) $$E\left(\hat{H}-H\right)=-\frac{k-1}{2n}+\frac{1}{12n^{2}}\left(1-\sum_{i=1}^{k}\frac{1}{p_{i}}\right)+O\left(n^{-3}\right),$$

and

(9) $$Var\left(\hat{H}\right)=\frac{1}{n}\left(\sum_{i=1}^{k}p_{i}\;ln^{2}\left(p_{i}\right)-H^{2}\right)+\frac{k-1}{2n^{2}}+O\left(n^{-3}\right),$$

where *n* is the sample size. If the bias’s expression terms that include unknown parameters are depreciated, then the bias is calculable when the cardinality of the alphabet is known, as in this case, but the variance is not since it depends on the unknown probabilities $p_{i}$. In this work, the point estimation of the mean μ=E(Q) and the variance $\sigma^{2}=V\left(Q\right)$ of the *Q* statistic was carried out directly, using the expressions

(10) $$\hat{\mu }=\hat{E(Q)}=\sum_{t=0}^{T}\hat{E(H_{z}^{t})},$$

and

(11) $$\hat{\sigma^{2}}=\hat{V(Q)}=\sum_{t=0}^{T}\hat{V(H_{z}^{t})}$$

respectively, based on the point estimation [26] of the means $\hat{E(H_{z}^{t})}$ and the variances $\hat{V(H_{z}^{t})}$ of each entropy $H_{z}^{t}$ for each time t, with t=1,…,T.

The lower the value of the *Q*-statistic, the less uncertainty about *j*, and, therefore, the sample would be more vulnerable to these attacks. To evaluate a set of samples of equal length, it is enough to calculate the test statistic for them and sort them increasingly. To compare samples of different lengths, the statistics obtained can be divided between the lengths of their respective samples, obtaining the average uncertainty per symbol and comparing in the same way.

### 3.3. Decision Criteria Using the *Q*-Statistic

The *Q*-statistic is defined as the sum of *T* random variables $H_{z}^{t}$. Following the results obtained in [23] by Zhang and Zhang, the plug-in entropy estimator, used to estimate the entropy considered in this work, follows an approximately normal distribution. In this way, assuming independence between the random variables $H_{z}^{t}$, the *Q*-statistic follows a normal distribution $N\left(\mu ,\;\sigma^{2}\right)$ with mean μ and variance $\sigma^{2}$ because the sum of Normal independent variables is also normal. Then, it is possible to approximate the distribution of the *Q*-statistic to a random variable with standard normal distribution.

(12) $$Q_{01}=\frac{Q-\mu }{\sigma }∼N\left(0,1\right),$$

where σ is the standard deviation of *Q*.

As mentioned above, the permutation of RC4 has biases that are transferred to *j* and the output *z*. The appearance of biases in the distribution $P(j_{t}|z_{t})$ provides alterations in the values of $H_{z}^{t}$ and consequently in the distribution of the *Q*-statistic, leading to the appearance of extreme values. The appearance of these extreme values adds to the distribution of *Q* a slight asymmetry on left tail since the alteration in the distribution of $P(j_{t}|z_{t})$ decreases the value of $H_{z}^{t}\;$ and, therefore, Q≪μ. For this reason, we will work with the standard normal distribution N(0,1) with a single tail, in this case with a left tail, and using a significance level α, it is concluded that the sequences from RC4 that provides more information about the variable *j* of the internal state are those with the lowest value of *Q*, such that $Q_{01}<Z_{\alpha }$.

## 4. Pre-Computing of Probabilities and Estimation of Entropies

The proposed method is divided into two phases, following an idea similar to a time memory trade off (TMTO) attack [27]. The first is the precomputation phase, often called the offline phase, where the probabilities and entropy in each time of *T* are estimated over each output value $z_{t}$. The objective of this phase is to estimate the information, in general, that provides the output occurrence $z_{t}$ on the variable $j_{t}$. This phase is executed only once, and then used repeatedly in the next phase for the evaluation of *N* outputs of the RC4. Although the second is referred to as the real-time or online phase, where it captures a sample of RC4 keystream and checks if this happens to be in the tables below. Each of the *M* outputs were generated from initializing the RC4 with *M* random inputs of 20 bytes each.

To estimate the conditional probabilities $P(j_{t}|z_{t})$, at each time t∈T for all possible values $z_{t}$, a pre-calculation of frequencies was performed, and thus the entropies were estimated.

To make a good estimation of the probabilities, in this work, we used *M* = 262,144,000 outputs of RC4 to reliably obtain as many biases as possible that RC4 has and taking into account the size k=256 of the alphabet.

### 4.1. Frequency Pre-Calculation

To calculate the frequencies, *M* = 262,144,000 outputs of the RC4 of length T=512 were generated and, at each time t∈T, the value of the pair $\left(j_{t},z_{t}\right)$ was checked, obtaining for each fixed $z_{t}$ the joint distribution $\left(j_{t},z_{t}\right)$ varying $j_{t}$. The value of *M* was chosen in order to obtain an expected frequency of

(13) $$E\left(f_{\left(j_{t},z_{t}\right)}\right)=262,144,000/\left(256\times 256\right)=4000$$

observations, by category, under the hypothesis of equi-probability.

A matrix of 256×256 was obtained for each time t=1,…,512 which represents each value of $z_{t}=0,\ldots ,255$ per column and in the rows each value from $j_{t}=0,\ldots ,255$. Thus, we have in row *j*, column *z*, the frequency $f_{\left(j_{t},z_{t}\right)}$ of joint appearance of the pair $\left(j_{t},z_{t}\right)$ at time *t* (see Table 1).

### 4.2. Estimation of Joint, Marginal, and Conditional Probabilities

From the joint frequencies $f_{\left(j_{t},z_{t}\right)}$ we can obtain the marginal frequency $f_{\left(\;z_{t}\right)}$ at each time *t*, to estimate the joint probability $P(j_{t},z_{t})$ and the marginal probability $P(z_{t})$, in order to reach an estimate of the conditional probability $P(j_{t}|z_{t})$, through the Bayes formula

(14) $$\hat{P}\left(j_{t},z_{t}\right)=\frac{f_{\left(j_{t},z_{t}\right)}}{M},$$

(15) $$\hat{P}\left(z_{t}\right)=\frac{f_{\left(z_{t}\right)}}{M}=\frac{\sum_{j=0}^{255}f_{\left(j_{t},z_{t}\right)}}{M},$$

and thus

(16) $$\hat{P}\left(j_{t}|z_{t}\right)=\frac{\hat{P}\left(j_{t},z_{t}\right)}{\hat{P}\left(z_{t}\right)}=\frac{\frac{f_{\left(j_{t},z_{t}\right)}}{M}}{\frac{\sum_{j=0}^{255}f_{\left(j_{t},z_{t}\right)}}{M}}=\frac{f_{\left(j_{t},z_{t}\right)}}{\sum_{j=0}^{255}f_{\left(j_{t},z_{t}\right)}}.$$

From the estimation of these probabilities, a table like the Table 2 is obtained, which now contains the conditional probability $\hat{P}\left(j_{t}|z_{t}\right)$ for each time t=1,…,512.

### 4.3. Entropy Estimation

For each time t∈T, the entropy $H_{z}^{t}=-\sum_{j_{t}=0}^{255}P\left(j_{t}|z_{t}\right)logP\left(j_{t}|z_{t}\right)$ was estimated, using the plug-in estimator [28]. This constitutes the entropy of the distribution of the *j* conditioned to the value $z_{t}$ of that column. Thus, at each time t∈T, 256 values of ${\hat{H}}_{z}^{t}$ are obtained. The output $z_{t}$ with the highest entropy ${\hat{H}}_{z}^{t}$ (tighter distribution to the uniform) provides the less information on *j*. Uniting the results obtained for the T=512 times, a matrix of 256×512 is obtained which contains per column each value of t=1,…,512 and in the rows each value of z=0,…,255. In each category (z,t) will be the entropy value ${\hat{H}}_{z}^{t}$ corresponding to row *z* and column *t* (see Table 3).

Then, to evaluate a particular sample, the value ${\hat{H}}_{z}^{t}$ corresponding to the place $\left(t,z_{t}\right)$ of the matrix is added using the statistic at each time *t*. In this way, a random variable of the type is obtained at each time

(17) $$\left(\begin{array}{ccc} {\hat{H}}_{z=0}^{t} & \ldots & {\hat{H}}_{z=255}^{t}\\ P\left(z_{t}=0\right) & \ldots & P\left(z_{t}=255\right) \end{array}\right),$$

whose expected value will be:

(18) $$E\left[{\hat{H}}_{z}^{t}\right]=\sum_{i=0}^{255}\hat{P}\left(z_{t}=i\right)\cdot {\hat{H}}_{z}^{t}\;={\hat{H}}^{t}\left(J/Z\right),$$

which constitutes the average uncertainty over *j*, at time *t*, when $z_{t}$ is known, i.e., the conditional entropy.

## 5. Experimental Evaluation

**Figure 1:**
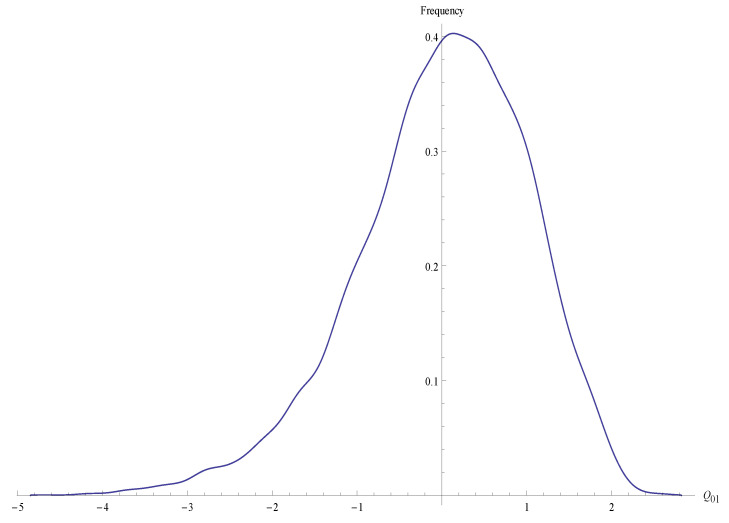
Distribution of $Q_{01}$ values in the 10,000 samples generated.

In the experiments ran for the present article, T=512 times will be taken, as in the pre-calculation stage and N=10,000 output sequences of the RC4 were generated from *N* random entries of 20 bytes each. The *T* value can be a variable parameter depending on the size required for the sample given the pre-calculation performed. For higher value selection of this parameter, it is necessary to deepen the theoretical comparison between the times and carry out more experiments. Figure 1 shows the distribution of the *Q*-statistic calculated at the 10,000 sequences generated. The left skewness illustrates the appearance of biases in the $P(j_{t}|z_{t})$ distribution that decrease the value of *Q*.

**Figure 2:**
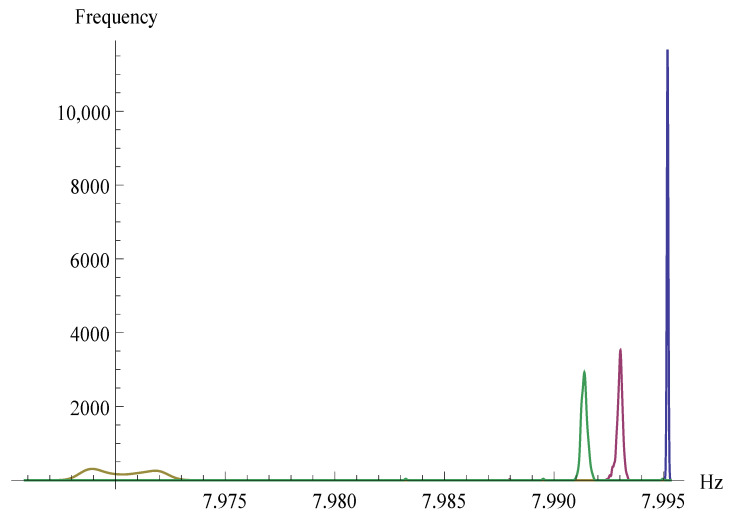
Representation of the extreme values of $H_{z}^{t}$ in the pre-calculation stage.

These biases are represented through the appearance of extreme values in each sequence. Figure 2 shows the extreme values of the pre-calculated $H_{z}^{t}$ distribution that cause such skewness to illustrate this event.

As can be seen, three groups stand out to the left of extreme values. The first two groups of extreme values are caused by the first and second output bytes of RC4, which are highly biases and not evenly distributed [21,22]. Then a third group, which is caused by the existence of $z_{t}$ bytes in the rest of the outputs, with t>1, that has a high correlation with *j*. The last group is the remaining values of $H_{z}^{t}$.

Finally, for a significance level α=0.01, it was obtained that 233 of the 10,000 sequences of outputs analyzed do not satisfy that $Q_{01}>Z_{\alpha }=-2326$. In other words, the output sequences that provide more information about the *j* variable were detected. In this way, the *Q*-statistic is able to distinguish within a set of RC4 output sequences the most vulnerable to iterative probabilistic attacks.

## 6. Conclusions

A statistical criterion was proposed, which allows for distinguishing a set of sequences of outputs of RC4. This *Q*-statistic is based on the conditional entropies of $j_{t}$ given the value $z_{t}$, known at each time *t*. It was experimentally verified that the proposed criterion could determine the existence of a class of output sequences more vulnerable to iterative probabilistic attacks. Future work intends to strengthen the proposed criterion by using the conditional probabilities $P(S_{t}|z_{t})$, as well as to extend the criterion to the case in which the output of RC4 is not known, and only the ciphertext obtained with that output is known. Another result will be to investigate the possible adjustment of the distribution of the *Q* statistic to some of the known distributions and theoretically determine the lowest value of M for which it is effective.

## Figures and Tables

**Table 1:** Frequency of joint appearance.

J/Z	0	1	…	255	
0	f(0,0)	f(0,1)	…	f(0,255)	$f_{\left(j_{t}=0\right)}$
1	f(1,0)	f(1,1)	…	f(1,255)	$f_{\left(j_{t}=1\right)}$
⋮	⋮	⋮	…	⋮	⋮
255	f(255,0)	f(255,1)	…	f(255,255)	$f_{\left(j_{t}=255\right)}$
	$f_{\left(z_{t}=0\right)}$	$f_{\left(z_{t}=1\right)}$	…	$f_{\left(z_{t}=255\right)}$	M

**Table 2:** Probabilities of *j* conditional on *z* at each time *t*.

J/Z	0	…	$z_{t}$	…	255
0	⋮				
⋮			⋮		⋮
$j_{t}$			$\hat{P}\left(j_{t}/z_{t}\right)$		
⋮	⋮		⋮	…	⋮
255					

**Table 3:** Entropy of the *j* distributions conditional on *z* at each time.

Z/T	1	2	…	512
0	${\hat{H}}_{0}^{1}$	${\hat{H}}_{0}^{2}$	…	${\hat{H}}_{0}^{512}$
1	${\hat{H}}_{1}^{1}$	${\hat{H}}_{1}^{2}$	…	${\hat{H}}_{1}^{512}$
⋮	⋮	⋮	…	⋮
255	${\hat{H}}_{255}^{1}$	${\hat{H}}_{255}^{2}$	…	${\hat{H}}_{255}^{512}$

## Data Availability

Data sharing not applicable.
